# Long-Term Outcome of Centrally Located Hepatocellular Carcinomas Treated by Radical Resection Combined With Intraoperative Electron Radiotherapy (IOERT)

**DOI:** 10.3389/fonc.2022.773301

**Published:** 2022-02-11

**Authors:** Yan-Ling Wu, Yirui Zhai, Minghui Li, Jian-Qiang Cai, Pan Ma, Li-Ming Wang, Xiu-Hong Wu, Xiao-dan Wang, Fan Wu, Qiang Zeng, Bo Chen, Ye-Xiong Li, Jian-Xiong Wu, Qinfu Feng

**Affiliations:** ^1^ Department of Radiation Oncology, National Cancer Center/National Clinical Research Center for Cancer/Cancer Hospital, Chinese Academy of Medical Sciences (CAMS) and Peking Union Medical College (PUMC), Beijing, China; ^2^ Department of Hepatobiliary Surgery, National Cancer Center/National Clinical Research Center for Cancer/Cancer Hospital, Chinese Academy of Medical Sciences (CAMS) and Peking Union Medical College (PUMC), Beijing, China; ^3^ Department of Operating Room, National Cancer Center/National Clinical Research Center for Cancer/Cancer Hospital, Chinese Academy of Medical Sciences (CAMS) and Peking Union Medical College (PUMC), Beijing, China

**Keywords:** centrally located hepatocellular carcinoma (CL-HCC), intraoperative electron radiotherapy (IOERT), safety, prognosis, narrow-margin resection

## Abstract

**Purpose:**

To explore the feasibility and safety of centrally located hepatocellular carcinoma (CL-HCC) treated by narrow-margin resection combined with intraoperative electron radiotherapy (IOERT).

**Methods and Materials:**

From November 2009 to November 2016, 37 consecutive patients were treated with IOERT as adjuvant treatment during narrow-margin resection for CL-HCC. Long-term outcomes, adverse events for surgery, and acute and chronic toxicities were analyzed.

**Results:**

The median follow-up was 57.82 months (range, 3.75-111.41 months). A total dose of 15 Gy (range 12 to 17Gy) (prescribed at the 90% isodose) was delivered with a 0.9cm (range 0.8-1.2 cm) median treatment depth targeting the narrow-margin. The 1-year, 3-year and 5-year OS rates were 91.39%, 88.34% and 88.34%, respectively. The 1-year, 3-year and 5-year DFS rates were 80.81%, 68.59% and 54.17%, respectively. In the univariate analysis, none of the treatment characteristics were predictive of overall survival. Fifteen (40.5%) patients suffered from a recurrence event. No patient had marginal recurrence. The 1-year, 3-year and 5-year intrahepatic recurrence rates were 19.75%, 25.92% and 39.58%, respectively. The 1-year, 3-year and 5-year extrahepatic recurrence rates were 2.7%, 5.95% and 9.87%, respectively. There was no 30-day surgical-related death. Three patients had grade 4, and 28 patients had grade 3 alanine aminotransferase (ALT) levels, and seven patients had grade 4, and 30 patients had grade 3 aspartate transaminase (AST) levels. All of them returned to normal within four months. There was no acute radiation-induced liver injury during follow-up. There were no acute or chronic toxicities associated with IOERT.

**Conclusion:**

IOERT for narrow-margin CL-HCC may achieve good long-term survival outcomes, without significantly increasing acute and chronic toxicities. An IOERT dose of 15Gy may be the safest and most feasible. IOERT might be considered as an adjuvant therapy for CL-HCC patients with a narrow-margin.

## Introduction

Hepatocellular carcinoma (HCC) is the third cause of cancer-related deaths worldwide. The incidence and mortality of HCC in China ranks the first in the world, and accounts for approximately half of all new HCC cases (1). Surgical resection is the main treatment method for HCC patients when liver transplantation is unavailable (2). However, long-term outcomes remain poor due to a high recurrence rate (5-year recurrence rate of nearly 70%) (3).

Centrally located hepatocellular carcinoma (CL-HCC) is a tumor located at the bifurcation of the portal vein, the confluence of the three main hepatic veins, the inferior vena cava or within 1 centimeter (cm) of the posterior inferior vena cava trunk. Due to its proximity to important blood vessels and bile ducts, ensuring a safe surgical resection margin of 1 cm is difficult. Narrower resection margins (<1 cm) and even null margins after surgery greatly increase the possibility of recurrence after surgery. It is reported that the recurrence rate of central liver cancer after five years is more than 90%, and the five-year DFS is 15-30% (4, 5). It is therefore paramount, to find efficacious adjuvant therapies that reduce CL-HCC recurrence.

HCC is sensitive to radiation with a sensitivity equivalent to squamous cancer cells. The local control rate of HCC is 71-100% after radical radiotherapy (6–9). Considering that the tumor bed tissue is at the highest risk of residual subclinical tumor cells, a local dose escalation has been shown to decrease CL-HCC recurrence rates most effectively (7). Intraoperative electron radiotherapy (IOERT) delivered immediately after surgery can reduce the spread of cancer cells. Direct visualization during surgery guarantees an accurate dose delivery to the tumor bed and provides greater protection of the normal tissue. In contrast to external-beam radiation therapy (EBRT), a single high-dose of radiation produces a higher biological effect and has the advantages of eliminating the residual micro-lesions in the tumor bed, and at the same time, intra- and interfractional motion is reduced and the dose applied more homogeneously. This technique provides more patient comfort and reduces total treatment time by one to five weeks (10–18).

IOERT as an adjuvant therapy has been used to treat head and neck, breast, prostate and gastrointestinal cancers, as well as retroperitoneal and extremity sarcomas (10–18). However, the application of IOERT is not well understood in CL-HCC patients. It is reasonable to hypothesize that IOERT may improve local control, and therefore, survival in CL-HCC patients. This study assesses the long-term survival and safety among CL-HCC patients who received adjuvant IOERT.

## Materials and Methods

### Patient Selection

This pilot study was approved by the ethics committee of the Cancer Hospital of the Chinese Academy of Medical Sciences (NCC2013RE-079). The inclusion criteria were: (1) two kinds of imaging examinations or pathologically confirmed CL-HCC prior to surgery; (2) no preoperative radiofrequency, ablation, intervention or chemoradiotherapy; (3) single or multiple lesions limited to three liver segments and able to be completely resected; (4) a resection margin of <1 cm; (5) no residual tumor with intraoperative frozen pathology, and enough liver tissue to ensure function; (6) an Eastern Cooperative Oncology Group (ECOG) score ≤1, a Child-Pugh score of A, ICG clearance (ICG. R 15) < 15%; and (7) no heart, lung, kidney, or other serious surgical complications.

From October 2009 to November 2016, the clinical records of 37 consecutive patients diagnosed with CL-HCC treated with IOERT as adjuvant treatment during narrow-margin resection were retrospectively reviewed in this prospectively assembled database. The database tracks patient medical history, family history, comorbidities, clinical history, tumor characteristics, diagnostic tests, therapeutic interventions, complications, and outcomes.

### Treatment

Pretreatment evaluation included medical history and physical examination, serum biochemistry for liver function status, clear imaging of the tumor, blood vessels and bile duct with pelvis multiphase contrast-enhanced computed tomography (CT), magnetic resonance imaging (MRI) or ultra­sound. Surgery and radiotherapy experts identified those suitable for IOERT. Patients were informed of the possible benefits and potential risks. All patients provided written informed consent.

### Surgery

Preoperative examination and an exploratory laparotomy were conducted to assess whether the tumor had metastasized, the degree of hepatic cirrhosis, tumor location, tumor size, the relationship between the tumor, important blood vessels and the bile ducts. Ultrasound examination was used to diagnose portal vein or major artery invasion. The extent of resection included the tumor and at least 1cm of the surrounding tissue to achieve mesohepatectomy, two- or three-segment resection, segmentectomy or non-anatomical hepatectomy according to the National Comprehensive Cancer Network guidelines (19).

Intraoperative ultrasonography and a cavitron ultrasonic surgical aspirator were used to carefully dissect and peel away the tumor from the major vascular surface. If resection of the major vessels was unavoidable, revascularization was performed to preserve more liver tissue and prevent postoperative liver failure. After the tumor was completely resected, the frozen resection margin was sent for a quick pathological examination to confirm there was no residual tumor before starting IOERT. Following IOERT, the abdomen was closed after the confirmation of no active liver bleeding, biliary fistulas and normal color of the remaining liver.

### Intraoperative Electron Radiotherapy

IOERT was initiated after confirmation of the target volume by radiotherapists and surgeons. The resected margin receiving IOERT, only extended to the high risk area near to the major vasculature, duct structures and the deep resected margin. The surface resection margins were at a sufficient safety distance. A smaller target volume reduced the possibility of radiation-induced liver damage. The desired IOERT field size and depth were achieved via the most appropriate angle and diameter of the applicator tube, electron beam energy, and the covering of bolus while minimizing the exposure of the adjacent normal tissue.

**Figure 1:**
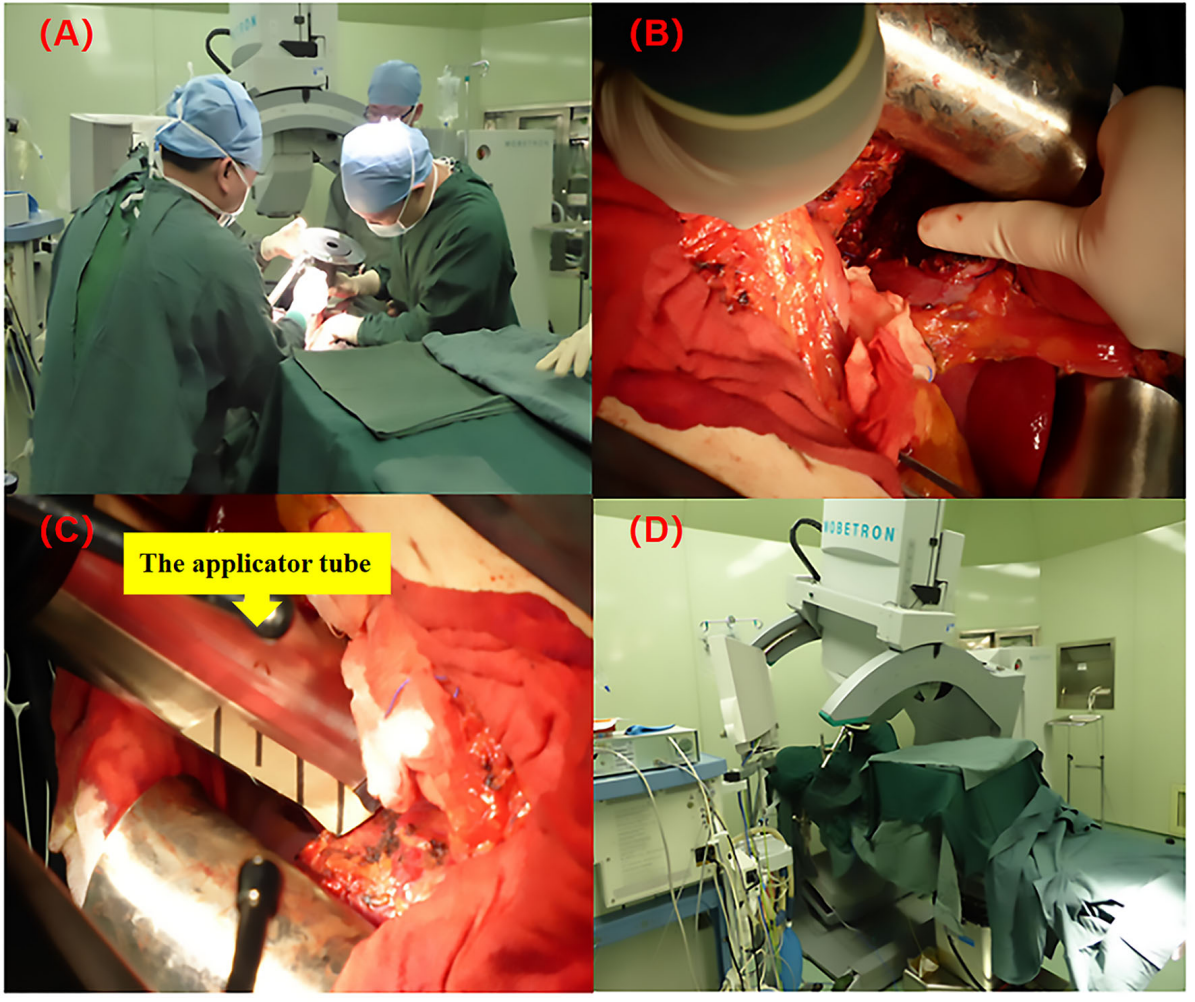
The IOERT process using the Mobetron 2000 mobile electron accelerator. **(A)** Radiotherapists and surgeons are confirming the target volume of IOERT; **(B)** The resected margin receiving IOERT, only extended to the high risk area near to the major vasculature, duct structures and the deep resected margin (the direction of the finger); **(C)** The applicator tube was placed under visual control (the diameter of applicator tube was ranged from 3 to 10 cm, and was available in 0.5 cm steps with 0°, 15°, and 30° angles); **(D)** IOERT was administered using a Mobetron 1000 mobile intraoperative radiotherapy accelerator (Intraop Medical Corporation, Sunnyvale, CA).

The applicator tube was placed under visual control and ranged from 3 to 10 cm in diameter, and was available in 0.5 cm steps with 0°, 15°, and 30° angles. High energy electron (6/9 MeV) beam radiotherapy was delivered with a total dose of 15 Gy (range from 12 to 17Gy) (prescribed at the 90% isodose) with a 0.9cm (range, 0.8-1.2 cm) median treatment depth targeting the narrow-margin. IOERT was administered using a Mobetron 1000 mobile intraoperative radiotherapy accelerator (Intraop Medical Corporation, Sunnyvale, CA) in the operating room (See **Figure 1**).

### Toxicity Criteria

Acute toxicity was evaluated one month after IOERT. Chronic toxicity was defined as a morbidity occurring at least one month after IOERT. Acute and chronic radiation toxicities were evaluated according to the Radiation Therapy Oncology Group (RTOG) and the European Organization for Research and Treatment of Cancer (EORTC) common Toxicity Criteria, version 4.0 (CTCAE 4.0) (Cancer Therapy Evaluation Program, Common Terminology Criteria for Adverse Events, Bethesda, MD, USA).

Radiation-induced liver disease (RILD) was defined as having an elevated anicteric alkaline phosphate level at least two times of the pre-treatment level and non-malignant ascites (classic RILD) (20), or, elevated transaminases at least five times the pre-treatment level (non-classic RILD) (21) in the absence of documented progressive disease. Patients were evaluated for RILD four months after radiotherapy. RILD was excluded from liver injury caused by drug hepatitis, interventional therapy, an acute outbreak of viral hepatitis, obstructive jaundice, and intrahepatic tumor progression.

### Recurrence Criteria

Recurrence was evaluated by clinical follow-up and imaging. Abdominal ultrasound, enhanced CT and MRI were used to find new intrahepatic, extrahepatic nodules or features of tumor malignancy. If there was no tumor recurrence by imaging examination but the alpha-fetoprotein (AFP) was larger than 400 ug/L for more than four weeks or 200 ug/L for more than eight weeks after excluding for a reproductive system tumor, pregnancy, and hepatitis, a diagnosis of tumor recurrence was made. Recurrence was divided into intrahepatic and extrahepatic relapse, marginal recurrence, and early and late recurrence. The criteria for marginal recurrence were defined on whether recurrence was inside the 1 cm surgical margin area, since 0.9 cm was the median IOERT depth. Early recurrence was defined as within 18 months. Late recurrence was defined as 18 months or over (22).

### Follow-Up and Statistical Analysis

Follow-up was every three months during the first two years, and every six months thereafter. Follow-up included: routine blood work, biochemical, blood clotting and tumor markers and abdominal CT/MRI/B ultrasound analysis. Overall survival (OS) was calculated as the time from surgical resection to death of any cause. DFS was calculated as the time from surgical resection to intrahepatic or extrahepatic recurrence of CL-HCC.

Statistical analyses were performed using SPSS v22.0 (IBM Corp, Armonk, NY). The counted data were analyzed by the T test, and the X^2^ test. Survival rates were calculated using the Kaplan–Meier method and intergroup differences compared using the log-rank test. Prognostic factors for OS were evaluated at the univariate level using the Cox proportional hazards models. A two-sided P value <0.05 was considered statistically significant in all the analyses.

## Results

### Patients, Tumors and Treatment Characteristics

The median follow-up was 57.82 months (range, 3.75-111.41 months). Thirty-two patients (86.5%) were male. The median age was 58 years old (range, 37-75 years old). Risk factors included long-term drinking history (n = 5), HBV positive status (n = 27) and cirrhosis (n = 34). All had Child-Pugh grade A liver function. A tumor marker AFP value ≥ 400ug/L was present in 11 patients. Thirty-three patients had single lesions, and four patients had multiple intrahepatic lesions. According to the American Joint Commission on Cancer (AJCC, 7^th^ ed.) staging system for HCC, stage T1, T2 and T3 were recorded as 20 (54.1%), 13 (35.1%) and 4 (10.8%), respectively. Nine patients had tumor infiltration/compression of the major vasculature, nine patients had microvascular invasion of the tumor, and 22 patients had liver capsule invasion of the tumor.

The median operation time was 285 minutes (range, 195-510 minutes). One patient had pleural effusion after surgery which improved after thoracic puncture and drainage. One patient developed acute renal failure on the second day and received renal replacement therapy and returned to normal within one week. There was no pulmonary infection, abdominal infection, incision infection, massive bleeding, or death within 30 days of surgery. The median tumor diameter was 5cm (range, 2-15cm). Fourteen patients had a diameter >5cm and 23 patients ≤5cm. The median applicator diameter was 5.5cm (range, 3-10cm). The median IOERT dose was 15Gy (range, 12-17Gy), three patients received ≤ 15Gy, 23 patients received 15Gy, and 11 patients >15Gy. For patients’ baseline, tumors and treatment characteristics, see **Table 1**.

**Table 1:** Demographic and clinicopathological features of the 37 patients with radical resection combined with IOERT.

Variables	Level	No. of patients (%)
Age (years)	Median (min–max)	58 (37~75)
	≤ 60	23 (62.2)
	> 60	14 (37.8)
Gender	Male	32 (86.5)
	Female	5 (13.5)
Chronic hepatitis	HBV+	27 (73)
	Cirrhosis	34 (91.9)
Long-term drinker	yes	5 (13.5)
	no	32 (86.5)
AFP level (ug/L)	<400 ug/L	26 (70.3)
	≥400 ug/L	11 (29.7)
Tumor diameter size (cm)	≤5cm	23 (62.2)
	>5cm	14 (37.8)
Number of primary tumors	Single	33 (89.2)
	Multiple	4 (10.8)
Histopathology grade (WHO)	Well-Mid	26 (70.3)
	Poor	11 (29.7)
T stage (AJCC, 7th ed.)	T1	20 (54.1)
	T2	13 (35.1)
	T3	4 (10.8)
Tumor infiltration/compression of the major vasculature		9 (24.3)
Microvascular invasion of the tumor		9 (24.3)
Presence of microsatellites		0
Liver capsule invasion of the tumor		22 (59.5)
Intraoperative blood transfusion		12 (32.4)
Postoperative bile leakage		0
30-day operative mortality		0
The depth of IOERT (cm)	Median (min–max)	0.9 (0.8~1.2)
Applicator diameter for IOERT (cm)	Median (min–max)	5.5 (3~10)
	≤ 5cm	19 (51.4)
	>5cm	18 (48.6)
IOERT energy	6 MV	28 (75.7)
	9 MV	9 (24.3)
Cone applicator angle for IOERT	0°	2 (5.4)
	15°	7 (18.9)
	30°	28 (75.7)
IOERT dose	12-14 Gy	3 (8.1)
	15 Gy	23 (62.2)
	16-17 Gy	11 (29.7)
Use of bolus	1cm	12 (32.4)
	0.5cm	25 (67.6)

IOERT, Intraoperative Electron Radiotherapy; AFP, alpha-fetoprotein; AJCC, American Joint Committee on Cancer; WHO, World Health Organization; HBV, Hepatitis B virus.

### Survival Rates

**Figure 2:**
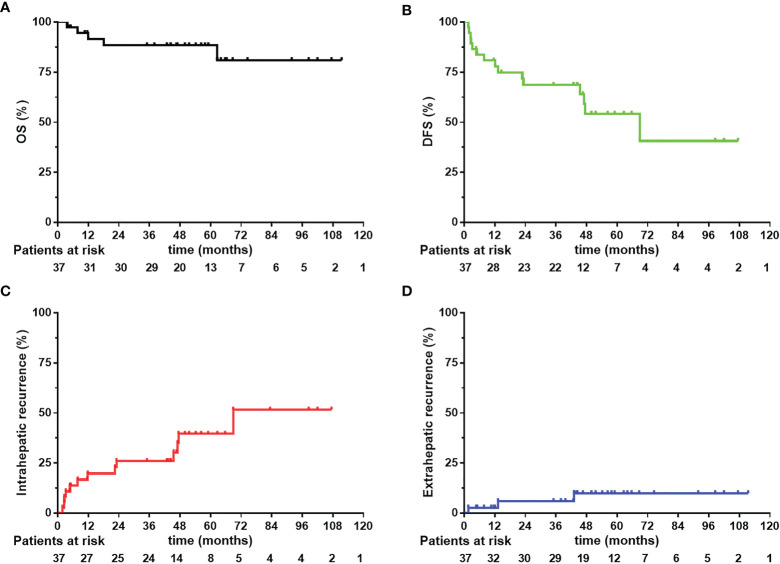
Overall survival (OS), disease-free survival (DFS) **(A, B)** and intrahepatic and extrahepatic recurrence curves **(C, D)** 37 CL-HCC patients received narrow-margin resection combined with IOERT. IOERT, Intraoperative Electron Radiotherapy.

By the end of the follow-up time, five patients (13.5%) were dead, four of which (10.8%) died from HCC related failure. Among them, three died from liver failure caused by intrahepatic recurrence and subsequent tumor-related treatment. One patient died from systemic multi-organ failure caused by multiple metastasis, and one patient with multiple bone metastasis who was stable after treatment died from respiratory failure due to poor lung function. The 1-year, 3-year and 5-year OS were 91.39%, 88.34% and 88.34%, respectively (see **Figure 2A**). The 1-year, 3-year and 5-year DFS were 80.81%, 68.59% and 54.17%, respectively (see **Figure 2B**).

### Univariate Analysis

We analyzed which patient characteristics and treatment parameters might be associated with survival outcomes (see **Table 2**). Among patient characteristics, the following factors entered the univariate survival analysis and demonstrated no statistical significance: age (P = 0.291), gender (P = 0.319), hepatitis B virus (HBV) (P = 0.372), long-term drinking (P = 0.693), AFP value (P = 0.237), tumor size (P = 0.17), histological grading (P = 0.076), staging (P = 0.818), tumor infiltration/compression of the vasculature (P = 0.931), tumor microvascular invasion (P = 0.116), and tumor liver capsule invasion (P=0.47). Among the treatment parameters, the diameter of the IOERT applicator tube (P = 0.057) and the IOERT energy (P = 0.255) were not statistically significant risk factors with survival outcomes.

**Table 2:** Univariate analysis for overall survival.

Prognostic factors	HR	95% CI	P-value
Age (years) (>60 *vs.* ≤60)	0.304	0.033-2.773	0.291
Gender (male *vs.* female)	0.312	0.032-3.086	0.319
HBV (positive *vs.* negative)	37.597	0.013-107956.335	0.372
Long-term drinker (yes *vs.* no)	1.559	0.172-14.105	0.693
AFP value (ug/L) (>400 *vs.* ≤400)	306.309	0.023-4047801.226	0.237
Tumor size (cm) (>5 *vs.* ≤5)	3.581	0.578-22.190	0.170
Histological grading (WHO) (well-moderate *vs.* poor)	0.195	0.320-1.185	0.076
Stage (AJCC, 7th ed.) (T2-3 *vs.* T1)	1.240	0.198-7.761	0.818
Tumor infiltration/compression of the vasculature (yes *vs.* no)	1.102	0.120-10.088	0.931
Microvascular invasion (yes *vs.* no)	4.379	0.695-27.589	0.116
Liver capsule invasion (yes *vs.* no)	0.444	0.049-4.018	0.470
Diameter cone applicator of IOERT (cm) (>5 *vs.* ≤ 5)	10.235	0.937-111.802	0.057
IOERT energy (16~17Gy *vs.* 15Gy)	3.287	0.423-25.539	0.255

IOERT, Intraoperative Electron Radiotherapy; AFP, alpha-fetoprotein; AJCC, American Joint Committee on Cancer; WHO, World Health Organization; HBV, Hepatitis B virus; CI, confidence interval; HR, hazard ratio.

### Patterns of Recurrence

During the follow-up, fifteen (40.5%) patients suffered from a recurrence event (see **Table 3**). No patient had marginal recurrence. Twelve patients had intrahepatic recurrence, including nine patients with a single relapse, and three patients with multiple relapse. Three patients had extrahepatic recurrence, including one patient with multiple systemic metastases, one patient had intrahepatic relapse and peritoneal metastasis, and one patient had multiple bone metastases. There were nine patients (24.3%) with early recurrence and six patients (16.2%) with late recurrence. Eleven (29.7%) patients received trans-arterial chemoembolization (TACE) therapy after recurrence. The 1-year, 3-year and 5-year intrahepatic recurrence rates were 19.75%, 25.92% and 39.58%, respectively (see **Figure 2C**). The 1-year, 3-year and 5-year extrahepatic recurrence rates were 2.7%, 5.95% and 9.87%, respectively (see **Figure 2D**).

**Table 3:** Pattern of recurrence.

Variable	No. of patients (%)
Total recurrence	15 (40.5)
Time to recurrence	
early recurrence (≤18 months)	9 (24.3)
late recurrence (>18 months)	6 (16.2)
Intrahepatic recurrence	12 (32.4)
marginal recurrence	0
single nodule	9 (24.3)
Multiple nodules	3 (8.1)
Extrahepatic recurrence	3 (8.1)
Treatment after recurrence	
TACE	11 (29.7)
RFA	1 (2.7)
Chemotherapy	1 (2.7)
Molecular targeted therapy	1 (2.7)
surgery	1 (2.7)

TACE, trans-arterial chemoembolization; RFA, radiofrequency ablation.

### Toxicity

**Figure 3:**
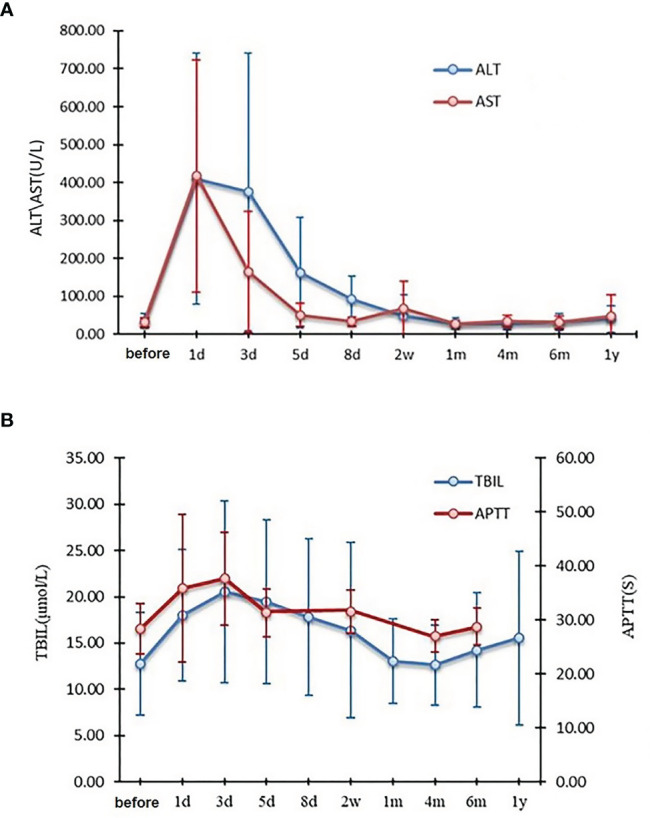
Changes in ALT and AST **(A)**, TBIL and APTT **(B)** before and after narrow-margin resection combined with IOERT treatment. IOERT, Intraoperative Electron Radiotherapy; ALT, alanine aminotransferase; AST, aspartate transaminase; TBIL, total bilirubin; APTT, activated partial coagulation time.

Renal electrolyte, coagulation, and liver function were collected and analyzed preoperatively and postoperatively on days 1, 3, 5, 8, and 14, and on months 1, 4, 6, 12 and 18. There were three patients with grade 4, and 28 patients with grade 3 alanine aminotransferase (ALT) levels, and seven patients with grade 4, and 30 patients with grade a 3 aspartate transaminase (AST) levels. All of them returned to normal within four months (**Figure 3**). Gamma-glutamyl transferase (GGT) and alkaline phosphatase (ALP/AKP) values were grade 1-2 and none for grades 3-4. All the other indicators were grade 1-2, except for one grade 3 patient with an activated partial coagulation time (APTT) who returned to normal within two weeks (**Figure 3**). None of the patients who received IOERT developed RILD. There were no acute or chronic toxicities associated with IOERT.

## Discussion

To the best of our knowledge, this is the first study to illustrate the long-term survival and safety of adjuvant therapy with IOERT for CL-HCC. Our data revealed that IOERT is a potentially viable adjuvant therapy for CL-HCC patients with a narrow-margin resection.

### Feasibility Considerations

Most studies have confirmed that insufficient margins can have a 16-50% recurrence rate (23–29). However, because the tumor is adjacent to major vessels for CL-HCC patients, narrow-margin resection is the only option, currently. CL-HCC with a RM <1cm has an unfavorable prognosis. Several studies have found that residual microscopic disease commonly occurs around the primary tumour (30–33). The main reason for early recurrence are residual microscopic lesions, a subclinical focus in the postoperative tumor bed, and peri-hepatic tumor minification metastasis by partial microvascular invasion (2, 3). Early recurrence is usually multi-focal or multi-nodule recurrence, so it is difficult to perform radical salvageable treatment (34). Therefore, the goal of reducing marginal and early recurrence may be achieved through microscopic lesion control. Effective adjuvant therapy targeting the narrow margin is the key to eradicating microscopic lesions around the main tumor.

Hepatocellular carcinoma cells have a high sensitivity to radiation, which could mean a similar control rate to squamous cell carcinoma is possible. The dosimetry advantages and clinical benefits of IOERT have been demonstrated among various malignancies (10–18). In our study there were 15 (40.5%) patients with recurrence, nine of which (24.3%) experienced early recurrence, but no patients had marginal recurrence compared with Yu W, et al., who reported a recurrence rate of 50.8% after resection alone (35). Similar results were also reported by Wang WH, et al., who showed that a narrow-margin resection for CL-HCC with a margin recurrence of 9.6% and an early recurrence of 43.4% (36). Although the results were compared in a single center, the treatment techniques and indications were similar and comparable. These studies indicate that narrow-margin resection combined with IOERT may reduce marginal and early recurrence.

Our study also shows favorable outcomes. The gain in survival is partially attributed to the reduction in marginal and early recurrence. In our study, the 1-year, 3-year and 5-year OS rates were 91.39%, 88.34% and 88.34%, respectively. The 1-year, 3-year and 5-year DFS rates were 80.81%, 68.59% and 54.17%, respectively. These results compare favorably to the results of Yu W, et al. during the same period and in the same hospital, with 74.5% and 40.1% for the 3-year OS and DFS using surgery alone, respectively (35). The 3-year OS and DSF increased by 13.84% and 28.49%, respectively. Wang WH, et al. retrospectively analyzed the results of 181 CL-HCC with IMRT in the same hospital. The results showed that the 3-year OS with a narrow-margin in the IMRT group, and surgery only group, and the wide-margin (> 1cm) group, were 89.1%, 67.7% and 86.0% (P =0.009), respectively, and the 3-year DFS was 64.2%, 52.2% and 60.1% (P=0.038), respectively. Postoperative radiotherapy may improve the effect in patients with narrow incisions (36). Based on these trials and our data, IOERT appears to control the residual tumor at the resection margin and persistent residual microscopic lesions in the remnant liver tissue surrounding primary HCC, which should decrease the risk of early intrahepatic and extrahepatic spread through the portal and hepatic veins. For patients at our institute with narrow margin hepatectomy, we recommend IOERT.

### Safety Considerations

While IOERT provides an opportunity for intraoperative adjuvant radiotherapy, it is difficult to preserve normal liver function. In our 37-patient study, most (95%) had multiple risk factors: fourteen patients had a tumor diameter >5cm, nine patients had tumor infiltration/compression of the major vasculature, nine patients had microvascular invasion of the tumor, and 34 patients had cirrhosis. Only one patient developed acute renal failure on the second day after surgery and returned to normal after one week in intensive care. Although there were changes in liver function before and after surgery, AST and ALT returned to normal within four months. There was no RILD found during follow-up.

Two small studies have reported on postoperative complications after IOERT adjuvant therapy. In a similar case control analysis by Xu C, et al. the liver function indicators in the two groups increased and recovered in a similar manner (P >0.05), without RILD occurence (37). Liu YH, et al. used a prospective cohort study to analyze the results of CL-HCC combined with IOERT, which was similar to our findings. With a prolonged surgical time, IOERT was safe and feasible (38). Taking all these findings into account, we conclude that IOERT adjuvant therapy does not increase the postoperative complication rate as was commonly thought. IOERT is safe as an adjuvant treatment for CL-HCC patients.

In the univariate analysis, none of the patients, tumor characteristics or IOERT parameters were predictive of overall survival. Since 2009, we have employed IOERT energy between 12 and 17Gy combined with adjuvant treatment without causing serious side effects. The current target volume and dose of IOERT may be suitable to improve tumor control despite the limited number of patients analyzed. We suggest that IOERT at 15Gy, at present, is the most appropriate dose as more energy does not appear to convey anymore benefits.

### Limitations

This was a retrospective cohort study using prospectively collected data, which minimized potential differential information bias. However, there are still some limitations. This was a single-institutional analysis. Therefore, the associated limitations need to be considered when interpreting these results. Besides, this pilot study included only a small number of patients receiving IOERT following narrow-margin resection for CL-HCC. It is difficult to adequately adjust for all potential confounding factors. However, the data does provide a rationale for developing well-designed clinical trials.

## Conclusions

This study revealed that IOERT as an adjuvant therapy for narrow-margin CL-HCC may achieve good long-term survival outcomes, without significantly increasing toxic side effects. It is suggested that an IOERT dose of 15Gy may be administered immediately after the tumor is resected with a surgical margin of approximately 1 cm. However, prospective studies with larger patient cohorts are needed to confirm these results.

## Data Availability Statement

The original contributions presented in the study are included in the article/supplementary material. Further inquiries can be directed to the corresponding author.

## Ethics Statement

The studies involving human participants were reviewed and approved by the ethics committee of the Cancer Hospital of the Chinese Academy of Medical Sciences. The patients/participants provided their written informed consent to participate in this study.

## Author Contributions

Contributorship statement study concepts: Y-lW, Y-xL, J-xW, Q-fF, and J-qC. Study design: Y-rZ, M-hL, J-qC, PM, L-mW, and X-hW. Data acquisition: Y-lW, Q-fF, FW. Quality control of data and algorithms: M-hL, J-qC, PM, L-mW, X-hW, J-xW, and Q-fF. Data analysis and interpretation: Y-lW, Y-rZ, J-xW, and Q-fF. Statistical analysis: M-hL, PM, L-mW, X-dW, FW, QZ, and BC. Manuscript preparation: Y-lW, M-hL, J-qC, and PM. Manuscript editing: Y-lW and Y-rZ. Manuscript review: Y-xL, J-qC, J-xW, and Q-fF. All authors contributed to the article and approved the submitted version.

## Funding

The study was supported by the Clinical Application Project of Beijing Municipal Science & Technology Commission (Z1107058811093), and the Beijing Municipal Science and Technology Project (Z131107002213166). The funders had no role in study design, data collection and analysis, decision to publish, or preparation of the manuscript.

## Conflict of Interest

The authors declare that the research was conducted in the absence of any commercial or financial relationships that could be construed as a potential conflict of interest.

## Publisher’s Note

All claims expressed in this article are solely those of the authors and do not necessarily represent those of their affiliated organizations, or those of the publisher, the editors and the reviewers. Any product that may be evaluated in this article, or claim that may be made by its manufacturer, is not guaranteed or endorsed by the publisher.

## References

[1] Siegel RL Fedewa SA Miller KD Goding-Sauer A Pinheiro PS Martinez-Tyson D. Cancer Statistics for Hispanics/Latinos, 2015. CA Cancer J Clin (2015) 65(6):457–80. doi: 10.3322/caac.21314 26375877

[2] Bruix J Sherman M. Management of Hepatocellular Carcinoma: An Update. Hepatology (2011) 53:1020–2. doi: 10.1002/hep.24199 PMC3084991 21374666

[3] Kobayashi T Ishiyama K Ohdan H. Prevention of Recurrence After Curative Treatment for Hepatocellular Carcinoma. Surg Today (2013) 43(12):1347–54. doi: 10.1007/s00595-012-0473-5 23271667

[4] Matsui Y Terakawa N Satoi S Kaibori M Kitade H Takai S. Postoperative Outcomes in Patients With Hepatocellular Carcinomas Resected With Exposure of the Tumor Surface, Clinical Role of the Nomargin Resection. Arch Surg (2007) 142:596–602. doi: 10.1001/archsurg.142.7.596 17638795

[5] Cheng CH Yu MC Wu TH Lee CF Chan KM Chou HS. Surgical Resection of Centrally Located Large Hepatocellular Carcinoma. Chang Gung Med J (2012) 35:178–91. doi: 10.4103/2319-4170.106153 22537933

[6] Rim CH Seong J. Application of Radiotherapy for Hepatocellular Carcinoma in Current Clinical Practice Guidelines. Radiat Oncol J (2016) 34(3):160–7. doi: 10.3857/roj.2016.01970 PMC5066447 27730805

[7] Hawkins MA Dawson LA. Radiation Therapy for Hepatocellular Carcinoma: From Palliation to Cure. Cancer (2006) 106:1653–63. doi: 10.1002/cncr.21811 16541431

[8] Krishnan S Dawson LA Seong J Akine Y Beddar S Briere TM. Radiotherapy for Hepatocellular Carcinoma: An Overview. Ann Surg Oncol (2008) 15:1015–24. doi: 10.1245/s10434-007-9729-5 18236114

[9] Jihye C Jinsil S. Application of Radiotherapeutic Strategies in the BCLC–defined Stages of Hepatocellular Carcinoma. Liver Cancer (2012) 1:216–25. doi: 10.1159/000343836 PMC3760456 24159586

[10] König L Lang K Heil J Golatta M Major G Krug D. Acute Toxicity and Early Oncological Outcomes After Intraoperative Electron Radiotherapy (IOERT) as Boost Followed by Whole Breast Irradiation in 157 Early Stage Breast Cancer Patients-First Clinical Results From a Single Center. Front Oncol (2019) 9:384. doi: 10.3389/fonc.2019.00384 31165041 PMC6536702

[11] Kaiser J Reitsamer R Kopp P Gaisberger C Kopp M Fischer T. Intraoperative Electron Radiotherapy (IOERT) in the Treatment of Primary Breast Cancer. Breast Care (Basel) (2018) 13(3):162–7. doi: 10.1159/000489637 PMC6062668 30069175

[12] Chen Y Che X Zhang J Huang H Zhao D Tian Y. Long-Term Results of Intraoperative Electron Beam Radiation Therapy for Nonmetastatic Locally Advanced Pancreatic Cancer. Med (Baltimore) (2016) 95(38):e4861. doi: 10.1097/MD.0000000000004861 PMC5044898 27661028

[13] Voogt ELK van Rees JM Hagemans JAW Rothbarth J Nieuwenhuijzen GAP Cnossen JS. Intraoperative Electron Beam Radiation Therapy (IOERT) Versus High-Dose-Rate Intraoperative Brachytherapy (HDR-IORT) in Patients With an R1 Resection for Locally Advanced or Locally Recurrent Rectal Cancer. Int J Radiat Oncol Biol Phys (2021) 110(4):1032–43. doi: 10.1016/j.ijrobp.2021.02.006 33567303

[14] Kyrgias G Hajiioannou J Tolia M Kouloulias V Lachanas V Skoulakis C. Intraoperative Radiation Therapy (IORT) in Head and Neck Cancer: A Systematic Review. Medicine (2016) 95(50):e5035. doi: 10.1097/MD.0000000000005035 27977569 PMC5268015

[15] Soriani A Landoni V Marzi S Iaccarino G Saracino B Arcangeli G. Setup Verification and *In Vivo* Dosimetry During Intraoperative Radiation Therapy (IORT) for Prostate Cancer. Med Phys (2007) 34:3205–10. doi: 10.1118/1.2750965 17879783

[16] Fu S Lu JJ Zhang Q Yang Z Peng L Xiong F. Intraoperative Radiotherapy Combined With Adjuvant Chemoradiotherapy for Locally Advanced Gastric Adenocarcinoma. Int J Radiat Oncol Biol Phys (2008) 72:1488–94. doi: 10.1016/j.ijrobp.2008.03.012 18538489

[17] Roeder F Alldinger I Uhl M Saleh-Ebrahimi L Schimmack S Mechtersheimer G. Intraoperative Electron Radiation Therapy in Retroperitoneal Sarcoma. Int J Radiat Oncol Biol Phys (2018) 100:516–27. doi: 10.1016/j.ijrobp.2017.10.034 29353660

[18] Roeder F de Paoli A Saleh-Ebrahimi L Alldinger I Bertola G Boz G. Intraoperative Electron Radiation Therapy Combined With External Beam Radiation Therapy After Gross Total Resection in Extremity Soft Tissue Sarcoma: A European Pooled Analysis. Ann Surg Oncol (2018) 25:3833–42. doi: 10.1245/s10434-018-6787-9 30276647

[19] Benson AB 3rd Abrams TA Ben-Josef E Bloomston PM Botha JF Clary BM. NCCN Clinical Practice Guidelines in Oncology: Hepatobiliary Cancers. J Natl Compr Canc Netw (2009) 7(4):350–91. doi: 10.6004/jnccn.2009.0027 PMC4461147 19406039

[20] Lawrence TS Ten Haken RK Kessler ML Robertson JM Lyman JT Lavigne ML. The Use of 3-D Dose Volume Analysis to Predict Radiation Hepatitis. Int J Radiat Oncol Biol Phys (1992) 23(4):781–8. doi: 10.1016/0360-3016(92)90651-W 1618671

[21] Trotti A Byhardt R Stetz J Gwede C Corn B Fu K. Common Toxicity Criteria: Version 2.0. An Improved Reference for Grading the Acute Effects of Cancer Treatment: Impact on Radiotherapy. Int J Radiat Oncol Biol Phys (2000) 47(1):13–47. doi: 10.1016/S0360-3016(99)00559-3 10758303

[22] Huang ZY Liang BY Xiong M Zhan DQ Wei S Wang GP. Long-Term Outcomes of Repeat Hepatic Resection in Patients With Recurrent Hepatocellular Carcinoma and Analysis of Recurrent Types and Their Prognosis: A Single-Center Experience in China. Ann Surg Oncol (2012) 19:2515–25. doi: 10.1245/s10434-012-2269-7 22395985

[23] Jeng KS Jeng WJ Sheen IS Lin CC Lin CK. Is Less Than 5 Mm as the Narrowest Surgical Margin Width in Central Resections of Hepatocellular Carcinoma Justifified? Am J Surg (2013) 206:64–71. doi: 10.1016/j.amjsurg.2012.06.010 23388427

[24] Chau GY Lui WY Tsay SH King KL Loong CC Chiu JH. Prognostic Signifificance of Surgical Margin in Hepatocellular Carcinoma Resection: An Analysis of 165 Childs’ A Patients. J Surg Oncol (1997) 66:122–6. doi: 10.1002/(SICI)1096-9098(199710)66:2<122::AID-JSO9>3.0.CO;2-F 9354168

[25] Hu RH Lee PH Chang YC Ho MC Yu SC. Treatment of Centrally Located Hepatocellular Carcinoma With Central Hepatectomy. Surgery (2003) 133:251–6. doi: 10.1067/msy.2003.102 12660635

[26] Ikai I Arii S Kojiro M Ichida T Makuuchi M Matsuyama Y. Reevaluation of Prognostic Factors for Survival After Liver Resection in Patients With Hepatocellular Carcinoma in a Japanese Nationwide Survey. Cancer (2004) 101:796–802. doi: 10.1002/cncr.20426 15305412

[27] Shi M Guo RP Lin XJ Zhang YQ Chen MS Zhang CQ. Partial Hepatectomy With Wide Versus Narrow Resection Margin for Solitary Hepatocellular Carcinoma: A Prospective Randomized Trial. Ann Surg (2007) 245:36–43. doi: 10.1097/01.sla.0000231758.07868.71 17197963 PMC1867934

[28] Nanashima A Sumida Y Abo T Nagasaki T Tobinaga S Fukuoka H. Comparison of Survival Between Anatomic and non-Anatomic Liver Resection in Patients With Hepatocellular Carcinoma: Signifificance of Surgical Margin in non-Anatomic Resection. Acta Chir Belg (2008) 108:532–7. doi: 10.1080/00015458.2008.11680280 19051461

[29] Hirokawa E Hayashi M Miyamoto Y Asakuma M Shimizu T Komeda K. Outcomes and Predictors of Microvascular Invasion of Solitary Hepatocellular Carcinoma. Hepatol Res (2014) 44:846–53. doi: 10.1111/hepr.12196 23834279

[30] Wang WH Feng X Zhang T Jin J Wang S Liu Y. Prospective Evaluation of Microscopic Extension Using Whole-Mount Preparation in Patients With Hepatocellular Carcinoma: Definition of Clinical Target Volume for Radiotherapy. Radiat Oncol (2010) 5:73. doi: 10.1186/1748-717X-5-73 20731853 PMC2936917

[31] Shi M Zhang CQ Zhang YQ Liang XM Li JQ. Micrometastasis of Solitary Hepatocellular Carcinoma and Appropriate Resection Margin. World J Surg (2004) 28:376–81. doi: 10.1007/s00268-003-7308-x 15022021

[32] Zhou XP Quan ZW Cong WM Yang N Zhang HB Zhang SH. Micrometastasis in Surrounding Liver and the Minimal Length of Resection Margin of Primary Liver Cancer. World J Gastroenterol (2007) 13:4498–503. doi: 10.3748/wjg.v13.i33.4498 PMC4611585 17724808

[33] Okusaka T Okada S Ueno H Ikeda M Shimada K Yamamoto J. Satellite Lesions in Patients With Small Hepatocellular Carcinoma With Reference to Clinicopathologic Features. Cancer (2002) 95:1931–7. doi: 10.1002/cncr.10892 12404287

[34] Zhang XF Beal EW Bagante F Chakedis J Weiss M Popescu I. Early Versus Late Recurrence of Intrahepatic Cholangiocarcinoma After Resection With Curative Intent. Br J Surg (2017) 105:848–56. doi: 10.1002/bjs.10676 29193010

[35] Yu W Wang W Rong W Wang L Xu Q Wu F. Adjuvant Radiotherapy in Centrally Located Hepatocellular Carcinomas After Hepatectomy With Narrow Margin (<1 Cm): A Prospective Randomized Study. J Am Coll Surg (2014) 218(3):381–92. doi: 10.1016/j.jamcollsurg.2013.11.030 24559953

[36] Wang WH Wang Z Wu JX Zhang T Rong WQ Wang LM. Survival Benefit With IMRT Following Narrow-Margin Hepatectomy in Patients With Hepatocellular Carcinoma Close to Major Vessels. Liver Int (2015) 35(12):2603–10. doi: 10.1111/liv.12857 25939444

[37] Xu C feng QF Bi XY Fan CC Zhai YR Li MH. Safety of Intraoperative Radiotherapy for HCC. Chin J Radiat Oncol (2014) 23(5):386–90. doi: 10.3760/cma.j.issn.1004-4221.2014.05.004

[38] Liu YH Wang LL Wu JH Rong WQ Wu F Li MH. A Prospective and Preliminary Study of Central Hepatocellular Carcinoma Combined With Intraoperative Tumor Bed Radiotherapy. Chin J Oncol (2017) 39(12):926–30. doi: 10.3760/cma.j.issn.0253-3766.2017.12.009 29262510

